# Chimeric antigen receptor-natural killer cell therapy: current advancements and strategies to overcome challenges

**DOI:** 10.3389/fimmu.2024.1384039

**Published:** 2024-04-25

**Authors:** Jun Chang Kong, Mohammad Auwal Sa’ad, Hema Manusri Vijayan, Manickam Ravichandran, Venugopal Balakrishnan, Seng Kong Tham, Gee Jun Tye

**Affiliations:** ^1^ Institute for Research in Molecular Medicine (INFORMM), Universiti Sains Malaysia, Minden, Penang, Malaysia; ^2^ Celestialab Sdn Bhd, Kuala Lumpur, Malaysia; ^3^ Department of Biotechnology, Faculty of Applied Sciences, AIMST University, Bedong, Kedah, Malaysia; ^4^ MyGenome, ALPS Global Holding, Kuala Lumpur, Malaysia; ^5^ ALPS Medical Centre, ALPS Global Holding, Kuala Lumpur, Malaysia

**Keywords:** chimeric antigen receptor (CAR), natural killer (NK) cell, advancements, strategies, challenges, cancer, immunotherapy

## Abstract

Chimeric antigen receptor-natural killer (CAR-NK) cell therapy is a novel immunotherapy targeting cancer cells via the generation of chimeric antigen receptors on NK cells which recognize specific cancer antigens. CAR-NK cell therapy is gaining attention nowadays owing to the ability of CAR-NK cells to release potent cytotoxicity against cancer cells without side effects such as cytokine release syndrome (CRS), neurotoxicity and graft-versus-host disease (GvHD). CAR-NK cells do not require antigen priming, thus enabling them to be used as “off-the-shelf” therapy. Nonetheless, CAR-NK cell therapy still possesses several challenges in eliminating cancer cells which reside in hypoxic and immunosuppressive tumor microenvironment. Therefore, this review is envisioned to explore the current advancements and limitations of CAR-NK cell therapy as well as discuss strategies to overcome the challenges faced by CAR-NK cell therapy. This review also aims to dissect the current status of clinical trials on CAR-NK cells and future recommendations for improving the effectiveness and safety of CAR-NK cell therapy.

## 1 Introduction

Adoptive cell therapy (ACT) is a promising immunotherapy targeting cancer cells by transferring specialized or engineered immune cells which are endowed with tumor-killing ability into a cancer patient (1). Among current ACTs implemented, chimeric antigen receptor (CAR) therapy becomes increasingly significant as T cells engineered with CAR (CAR-T) have shown clinical success in hematological malignancies in clinical settings owing to their remarkable anticancer abilities. Nevertheless, CAR-T therapy possesses several limitations such as tumor antigen loss, on target off tumor side effects, cytokine release syndrome (CRS) due to overwhelmed cytokines released during CAR-T cell activation, immune effector cell-associated neurotoxicity syndrome (ICANS), difficulty in trafficking and infiltrating into solid tumor cells, and immunosuppressive tumor microenvironment (2, 3). During CAR-T cell manufacture, large T cell populations are heavily required, thus surging the cost, and lengthening the manufacturing process which further burdens cancer patients, leading to rapid late-stage cancer progression (3, 4). Moreover, graft-versus-host disease (GvHD) is a major concern when using allogenic CAR-T cell therapy due to the presence of foreign donor antigens on T cells will stimulate the patient’s immune system (5).

To resolve these obstacles, current research is moving towards the production of CAR-NK cells on account of NK cells are innate immune cells which are human leukocyte antigen (HLA)-unrestricted and able to kill infected or cancerous cells without the need of antigen priming (6). Therefore, they are safe to be used as “off-the-shelf” therapy as no GvHD cases are reported so far (7).[NO_PRINTED_FORM]. Moreover, CAR-NK cells do not cause cytokine release syndrome (CRS) and neurotoxicity when compared to CAR-T cells. Different cytokines are released by CAR-NK cells to prevent CRS from happening (8). Furthermore, the specificity of antibody-based CAR ectodomain targeting specific tumor antigen and shorter lifespan of CAR-NK cells, limit the effect of on target off tumor toxicity (9).

NK cells in the blood can be categorized into two main populations based on the expression of cell surface receptors: CD56^bright^ CD16^dim^ NK cells and CD56^dim^ CD16^bright^ NK cells (10). Theoretically, CD56^bright^ CD16^dim^ NK cells (5% of total NK cells) are cytokine-producing cells residing in secondary lymphoid organs while CD56^dim^ CD16^bright^ NK cells (95% of total NK cells) are cytotoxic cells circulating in peripheral blood (11). The activation of NK cells is regulated based on the equilibrium of ligand stimulations on both activating and inhibitory receptors of NK cells (12). The activating and inhibitory receptors of NK cells play a pivotal role in signaling and eliminating infected or cancer cells.

Naturally, NK cells eliminate cancer cells via various molecular mechanisms. The downregulation of major histocompatibility complex (MHC) in cancer cells often leads to the failure of CAR-T cell therapy. However, the loss of MHC in cancer cells reduces the inhibitory signal on NK cells. Therefore, NK cells can still be activated and able to target cancer cells via the missing-self mechanism despite MHC being shed from cancer cells (13). Moreover, the aberrant overexpression of ligands on cancer cells can activate NK cells through an induced-self mechanism when the overexpressed ligands are bound to the activating receptors of NK cells, causing the induction of cytolytic activity in cancer cells (13). Furthermore, NK cells can eradicate cancer cells by recognizing non-self-antigens presented by the MHC of cancer cells (14). In addition, NK cells can bind their CD16 (FcγRIIIA) to target cancer cells which opsonized with IgG through antibody-dependent cell-mediated cytotoxicity (ADCC) (15). NK cells can trigger apoptosis of cancer cells via the Fas/FasL signaling pathway or TNF-related apoptosis-inducing ligand (TRAIL) pathway (16). Overall, CAR-NK cells employ these NK cell killing mechanisms, thus making them more robust and superior compared to CAR-T cells.

Nonetheless, CAR-NK cell therapy faces several challenges such as lack of persistence in CAR-NK cells, inhibition of CAR-NK cells due to inhibitory receptors on NK cells, immunosuppressive tumor microenvironment, ineffective CAR-NK cell trafficking and infiltration, and immune escape. In this review, current advancements and limitations of CAR-NK cell therapy are explored to improve and leverage their ability to eradicate and kill cancer cells. Lastly, we conclude this review by discussing strategies to overcome challenges and future recommendations on CAR-NK cell therapy.

## 2 Advancement of CAR-NK cell therapy

### 2.1 Optimizing CAR-NK structures

CAR-NK cells have been revolutionized over the years to explore the optimal CAR structure with enhanced cytotoxicity and better clinical responses for cancer patients. CAR-NK cells implement the concept of CAR-T cells, and thus develop into several generations. The first generation consists of an ectodomain, single chain fragment variable (scFv) linked with a transmembrane and CD3 endodomain. Nonetheless, the lack of a co-stimulating domain in the CAR structure leads to low proliferation of CAR-NK cells (12). Therefore, the second CAR generation tackles this problem by putting one co-stimulating domain such as DNAX activation protein (DAP) 10, DAP12, 2B4, and 4-1BB (CD137) into CAR structure (17). Future improvement has been done to enhance CAR structure through the addition of one more co-stimulating domain, thus generating the third CAR generation (17). To further improve the persistence and functionality of CAR-NK cells, the fourth generation of CAR is armored with cytokines such as IL-15 (18).

Optimizing CAR structure requires more novel strategies for CAR structural modification. Next generation of CAR-NK cells is the current trend to produce more effective and specific CAR, hoping to strengthen the tumor recognition ability of CAR-NK cells. Dual CAR NK cell which generates two independent receptors provides greater specificity in recognizing cancer cells. Cichocki et al. generated two independent anti-CD19 CAR and non-cleavable CD16 receptors on NK cells termed as iDuo NK cells offered higher specificity against CD19 cancer cells and prevented immune escape by targeting CD19-negative cancer cells (19). Cichocki et al. also showed that CD38 knockout CAR-NK cells which were transduced with dual targeting anti-BCMA CAR and CD16 possessed enhanced cytotoxicity and prolonged persistence against multiple myeloma cells (20). Recently, dual CAR-NK cells comprising anti-BCMA CAR and anti-GPRC5D demonstrated remarkable results in killing multiple myeloma cancer cells (21). To date, FT596, a muti-targeting CAR-NK cell which possesses anti-CD19 CAR, non-cleavable CD16 receptor, and IL15/IL-15α was developed to enhance cancer-killing ability and improve the persistence of CAR-NK cells (22). However, there are still few studies on synthesizing dual or multi-targeting CAR-NK cells to target cancer cells currently. Therefore, more preclinical studies should be done to explore dual or multi-targeting CAR-NK cells in eradicating solid tumors.

Bispecific CAR-NK cell is another advancement made for creating better CAR-NK cell immunotherapy. For instance, Kim et al. showed that bispecific anti-CD19-CD22 CAR-NK cells possessed anticancer ability to lyse B cell lymphoma effectively (23). Moreover, the synthetic Notch (synNotch) receptor which induces transcription factor upon antigen recognition is a promising approach to fine-tune the specificity of CAR-NK cells. NK cells transduced with synNotch receptor targeting GPC3 antigen of cancer cells were able to secrete IL-12 into the tumor microenvironment directly, thus aiding CAR-T cells to infiltrate into cancer cells (24). Furthermore, another approach is using inhibitory CAR to enhance CAR-NK cells in recognizing cancer cells without targeting healthy cells. For instance, a study of implementing inhibitory CAR on NK cells had successfully inhibited healthy NK cells which possessed trogocytic antigen from being killed by CAR-NK cells (25).

In addition, universal CAR shows a promising strategy to improve CAR-NK cell therapy. Mitwasi et al. developed universal CAR which could only be activated upon coupling with a specific target molecule, α-GD2 IgG4 to target GD2-positive cancer cells (26). Moreover, Kang et al. designed a universal modifiable cotinine-specific CAR which is bound to a cotinine conjugator to target multiple cancer antigens (27). Furthermore, the inducible caspase9 (iCasp9) suicide gene is used in on/off logic-gated CAR to switch off the killing mechanism of CAR-NK cells when needed (28).

## 3 Advancement of CAR-NK cell production

### 3.1 Exploring various NK cell sources

There are various sources to collect NK cells for CAR-NK cell production. One of the methods is from peripheral blood. Specifically, NK cells can be obtained either from autologous or allogenic peripheral blood. Nevertheless, the limited amount of autologous or allogenic NK cells obtained from peripheral blood not only impedes NK cell expansion, but also possesses heterogenous issues and is sensitive to freeze-thaw cycles which eventually reduces the efficiency of transduction (29). Therefore, another approach such as using NK cell lines is preferred to overcome the disadvantages of primary NK cells from peripheral blood.

CAR-NK cells can be engineered from cell line such as NK-92 cells due to this cell line possesses high cytotoxicity, short cell doubling time, lack of inhibitory receptors, and is easier to be genetically modified compared to other cell lines (30, 31). However, NK-92 cells possess several disadvantages such as a lack of CD16 receptors to mediate ADCC, the requirement for IL-2 supply to proliferate, and the need for irradiation to overcome the risk of immune rejection (30). Therefore, modifying NK-92 cells is highly demanded to resolve the limitations.

Up to date, there are a lot of advancements made for NK-92 cells. For instance, NK-92 cells are modified to produce IL-2-expressing NK cell lines such as NK-92ci and NK-92mi cells (31, 32). To solve the issue of lacking CD16 receptor, high-affinity Fc receptor expressing NK-92 cells (haNK) are produced by transducing NK cells with a plasmid carrying high-affinity CD16 and IL-2 (31, 32). Furthermore, haNK cells can also be used to express CAR, thus generating other new cell lines such as targeted high-affinity NK-92 cells (t-haNK) and quadrocistronic targeted high-affinity NK-92 cells (qt-haNK) to broaden the use of NK cell lines in cancer immunotherapy (31).

Apart from NK cell lines, stem cells are used to derive CAR-NK cells due to their ability to be used as “off-the-shelf” therapy. NK cells derived from umbilical cord blood possess higher proliferation capacity and can be easily obtained or frozen compared to peripheral blood NK cells (33). Most importantly, cord blood NK cells have better homing ability and are less immunogenic due to the higher chemokine receptor expression on cord blood-derived NK cells and lesser T cells isolated from cord blood (33). Nevertheless, NK cells from cord blood are immature and express lower expression of granzyme B and activating receptors such as CD16 and DNAM-1 (34). Furthermore, the higher expression of inhibitory receptors on cord blood NK cells hinders their function to kill cancer cells effectively (34).

In another perspective, NK cells derived from induced pluripotent stem cells (iPSCs) are the current advancement made for optimizing CAR-NK cell production. Although iPSCs-derived NK cells possess tumorigenic properties and laborious transdifferentiating procedures, NK cells from iPSCs are still a promising source to produce CAR-NK cells as they are consistently homogenous, highly proliferative, and can be scaled up to produce an unlimited supply of NK cells (35). Furthermore, the risk of GvHD is low in iPSCs-derived NK cells (36). Therefore, NK cells derived from iPSCs can be used as “off-the-shelf” therapy.

Overall, CAR-NK cells can be produced from various sources, thus highlighting the importance of exploring more strategies to synthesize even better “off-the-shelf” CAR-NK cell therapy for cancer patients. Moreover, more studies on exploring other NK cell sources should be investigated for optimizing and standardizing CAR-NK cell production.

### 3.2 Manipulating different CAR gene delivery methods

To deliver the CAR gene effectively into NK cells, various viral and non-viral methods have been implemented to produce CAR-NK cells. In fact, all methods possess pros and cons in delivering transgene. Therefore, it is hard to standardize the CAR gene delivery method and manufacture CAR-NK cells based on universal protocol. The pros and cons of respective gene delivery methods are tabulated in **Table 1**.

**Table 1:** Comparison between viral and non-viral vectors used for CAR gene delivery.

Aspect	Viral vector		Non-viral vector	
	Retrovirus	Lentivirus	PiggyBac transposon	Sleeping Beauty (SB)
Infection	Dividing cells	Dividing and Non-dividing cells	Dividing cells	Dividing cells
Transfer method	Transduction	Transduction	Electroporation	Electroporation
Gene Integration	Integrating	Integrating	Integrating	Integrating
Delivery efficiency	High	High	Moderate	Moderate
Packaging capacity	Limited size	Limited size	Large	Large
Expression stability	High	High	High	High
Immunogenicity	Moderate	Moderate	Low	Low
Safety	Risky	Risky	Risky	Risky
Cost	High	High	Low	Low
References	(37, 38)	(39)	(40, 41)	(40, 41)

Retrovirus and lentivirus are the common viral vectors used to delivery CAR gene effectively into NK cells while piggyBac transposon and sleeping beauty transposon are non-viral vector used for safer CAR gene delivery.

NK cells can be transduced by various viral and non-viral methods. One of the viral methods is using retrovirus. Although retrovirus is useful by integrating transgene into the host genome permanently, the risk of eliciting mutagenesis and only being able to transduce dividing NK cells limits the use of retrovirus in producing CAR-NK cells (37, 38). Therefore, other viral vectors such as lentiviral vectors are used to overcome the limitations of retroviral vectors. Lentivirus is commonly favored in delivering the CAR gene due to its ability to transduce dividing and non-dividing cells, permanently integrate transgene into the host genome, and low immunogenicity compared to other viral delivery methods (39). However, lentivirus may possess a risk of insertional mutation (39). Therefore, to ensure the safety of lentivirus in transgene delivery, second and third-generation lentiviral vectors are generated and widely used to produce CAR-NK cells. To date, one study on producing CAR-NK cells using second and third generations of lentiviral vectors showed no differences in transduction efficiency for both of them, thus highlighting both lentiviral vector generations were able to deliver CAR gene efficiency (42).

Apart from the viral method, recent studies on delivering the CAR gene via piggyBac transposons have been reported. Wang et al. used piggyBac transposons encoding NKG2D-DAP10 CAR to transfect NK cells against CD73-positive lung cancer cells (43). Wang et al. successfully demonstrated stable and sustained NKG2D expression in NK cells over the time of the study (43). Similarly, piggyBac transposon which co-expressed with IL-15 was used to generate CAR-NK cells targeting the NKG2D ligand of cancer cells (44). Therefore, these studies provide great insight into using non-viral piggyBac transposons to deliver the CAR gene which might offer safer clinical benefits for patients. Furthermore, non-viral vectors such as sleeping beauty (SB) can be used to transfect NK cells. Bexte et al. demonstrated that SB able to induce prolonged durability for CAR expression and robust cytotoxicity against acute lymphoblastic leukaemia (45). In fact, the use of piggyBac and SB transposons possess advantages such as large cargo size for transgene, low immunogenicity, and cheap manufacturing cost (40). Nonetheless, random transgene integration of piggyBac and SB transposons can lead to mutation or damage to the structure of the gene (41).

A new advancing method to deliver the CAR gene into NK cells is via clustered regularly interspaced short palindromic repeats and CRISPR-associated protein 9 (CRISPR-Cas9) technology. Using this technology, the CAR gene was carried by adeno-associated virus (AAV) and transfected into primary NK cells. The results showed that 68% of NK cells were successfully transfected to become CAR-NK cells (46, 47). CRISPR-Cas9 provides precise gene editing, but gene delivery efficiency still needs to be investigated (39).

### 3.3 Enhancing CAR transduction or transfection into NK cells

NK cells are resistant to being transduced or transfected (37, 38). Therefore, studies have been performed to increase transduction or transfection efficiency for NK cells. Enhancing viral transduction can be achieved by alternating electrical changes on the NK cell membrane. The use of a polycationic agent, polybrene showed enhanced NK cell transduction (89.2%) when compared to other enhancers for transduction such as vectofusin 1 (70.9%) and retronectin (30.4%) (48). Similarly, Kim et al. proved polybrene improved NK cell transduction, however, they also demonstrated that high polybrene concentration and prolonged duration of polybrene exposed to NK cells could decrease NK cell viability (49).

Moreover, lentiviral transduction can be improved by upregulating low-density lipoprotein receptor (LDLR) of NK cells to bind with the vesicular stomatitis virus (VSV) of lentiviral particles carrying the CAR gene. Gong et al. demonstrated a statin known as rosuvastatin was able to upregulate LDLR of NK cells, thus promoting better CAR transduction via the increase of NK cell binding with VSV of lentiviral particles (50). Furthermore, Baboon envelope pseudotyped lentiviral vectors (BaEV-LVs) expressed higher CAR expression on NK cells compared to other pseudotyped lentiviral vectors, thus enhancing the killing effects of CAR-NK cells towards NK-resistant blood cancer cells (51). A similar result was also observed in the study done by Soldierer et al. They also explored different types of promoters in regulating CAR gene expression, suggesting that the myeloproliferative sarcoma virus (MPSV) promoter was better than other promoters used (52).

In addition, NK cell transduction can be enhanced by incorporating TBK1/IKKϵ inhibitor with VSV-pseudotyped lentiviral vector. TBK1/IKKϵ inhibitor was used to inhibit toll-like receptor (TLR) 4 antiviral signaling pathway, thus enhancing the binding of VSV receptor with NK cells and eventually enhancing viral transduction (53).

From another perspective, advancement to improve transfection efficiency for non-viral CAR delivery method is through synthesizing a novel polymer to deliver the CAR gene. For instance, oligomer such as cationic charge-altering releasable transporters (CARTs) was synthesized by Wilk et al. to bind with anti-CD19 mRNA of CAR before encapsulating this CART-anti-CD19 mRNA in a polyplex to transfect NK cells (54). The transfection efficiency shown by this CART-anti-CD19 mRNA was higher than commercial transfection agents such as lipofectamine, thus providing a new and novel strategy to increase the transfection efficiency of non-viral methods into NK cells.

**Figure 1:**
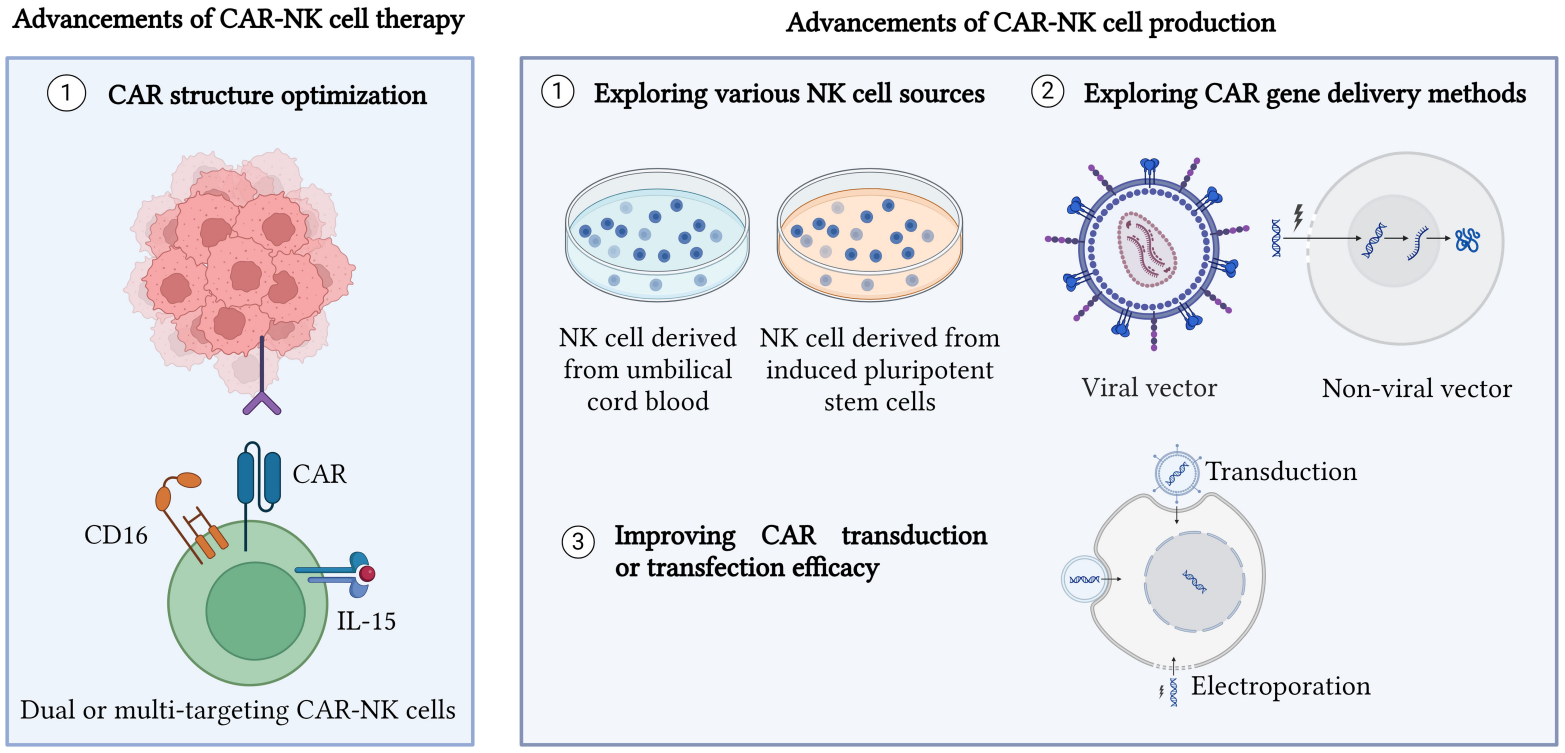
Advancements in CAR-NK cell therapy and production. CAR structure has been optimized to generate different generations of CAR as well as produce dual CAR-NK cells, bispecific CAR-NK cells, universal CAR-NK cells, and inhibitory CAR-NK cells. Moreover, exploring various new sources for NK cells such as umbilical cord blood and induced pluripotent stem cells (iPSCs) is also a new advancement made for CAR-NK cell therapy and production. Other approaches such as enhancing CAR gene delivery methods and improving CAR transduction or transfection efficiency are performed to improve CAR-NK cell production. Created with BioRender.com.

To facilitate CAR gene transfection via non-viral methods, other strategy such as nanoparticles is used to enhance the transfection efficiency. Douka et al. showed that lipid nanoparticles were superior compared to polymeric nanoparticles and electroporation in delivering messenger RNA (mRNA) carrying a green fluorescent transgene and ultimately promoting higher transfection efficiency for NK cells and T cells (55). Recently, Golubovskaya et al. proved lipid nanoparticles harboring mRNA of the CAR gene were successfully transfected into NK cells and killed cancer cells *in vitro* and *in vivo* (56). Therefore, lipid nanoparticles can be used to improve the transfection efficiency of CAR-NK cells. Furthermore, magnetic nanoparticles coated with polymer also showed improved transfection efficiency towards NK cells (57). However, more studies on lipid or magnetic nanoparticles in enhancing the transfection efficiency of NK cells should be performed to produce effective CAR-NK cells from non-viral methods. The advancements of CAR-NK cell therapy and production are illustrated in **Figure 1**.

## 4 Challenges of CAR-NK cell therapy

Although CAR-NK cell therapy shows promising potent anticancer results, it also possesses challenges which impair the efficacy of CAR-NK cells. One of the challenges is the lack of persistence in CAR-NK cells due to NK cells having shorter lifespans (3). Therefore, multiple rounds of CAR-NK cell administration may be required to maintain sufficient CAR-NK cells which have the potency to kill cancer cells in a patient’s body.

Moreover, NK cells express various inhibitory receptors such as killer cell immunoglobulin-like receptors (KIRs), NKG2A, PD-1, TIM-3, and TIGIT (58). Therefore, NK cells are easily inhibited and deactivated once their inhibitory receptors are bound with inhibitory ligands that are present in the tumor microenvironment.

Furthermore, CAR-NK cells are suppressed by hypoxic, nutrient-deficient, and immunosuppressive tumor microenvironment. The presence of myeloid-derived suppressor cells (MDSCs), cancer-associated fibroblasts (CAFs), regulatory T cells (Tregs), and tumor-associated macrophages (TAMs) suppress NK cell function, thus leading to inactivation of NK cells (59). NK cells are also suppressed by a nutrient-deficient environment due to a lack of glucose and amino acid supplies for them to survive (60). In addition, NK cells are hard to infiltrate into complex tumor microenvironment, thus leading to failure of CAR-NK cell therapy.

In another perspective, the loss of cancer antigens often leads to immune escape. One of the immune escape mechanisms is trogocytosis. Trogocytosis is an event involving the transfer of cancer antigens from cancer cells to NK cells, thus leading to antigen loss in cancer cells. NK cells which receive cancer antigens are targeted by CAR-NK cells (61). As a result, fratricide of NK cells occurs due to the NK cells present trogocytic antigen mediated by trogocytosis (61).

Additionally, NK cell exhaustion is also a main hurdle in CAR-NK cell therapy. The exhaustion of NK cells is mainly presented by several exhaustion phenotypes such as reduction in NK cell proliferation, activation, and cytotoxicity, downregulation of cytokines and activating receptors of NK cells, upregulation of immune checkpoints and inhibitory signals on NK cells, and dysregulation in NK cell metabolism (62). For instance, the upregulation of inhibitory receptors such as PD-1, TIGIT, TIM-3 on NK cells and increase in tumor-derived factors including prostaglandin E2 (PGE2), TGF-β, adenosine, and exosomes can eventually cause NK cell inhibition and exhaustion (62). Moreover, nutrient deprivation due to reduction in oxidative phosphorylation (OXPHOS) and glycolysis on NK cells can lead to NK cell exhaustion and cytotoxicity inhibition (63, 64). Furthermore, NK cells are exhausted by the hypoxic tumor microenvironment. Hypoxia will induce hypoxia-inducible factor 1-alpha (HIF-α) which can downregulate NK cell activating receptors and upregulate PD-L1 expression on immunosuppressive MDSCs, thus leading to NK cell inhibition (65, 66). In addition, lactate and lipid accumulation in tumor microenvironment as well as the presence of indoleamine 2,3-dioxygenase (IDO) show inhibition on NK cell proliferation, cytotoxicity release, and downregulation on NK cell activating receptors (67–69).

## 5 Strategies to tackle challenges in CAR-NK cell therapy

### 5.1 Improving activation, proliferation, and persistence of CAR-NK cells

The activation, proliferation, and persistence of CAR-NK cells are the major barriers to sustaining and stimulating sufficient CAR-NK cells to target cancer cells in a patient’s body. One of the strategies is supplying cytokines to stimulate NK cells. Cytokine such as IL-2 provides robust stimulation on NK cells, but IL-2 also activates suppressive T regulatory cells (Treg) which in turn impede NK cells to eliminate cancer cells (70). Therefore, Bentebibel et al. tackled this problem by using a promising human recombinant IL-2, namely bempegaldesleukin (NKTR-214) to boost the activation and expansion of immune cells such as NK cells and CD8^+^ T cells with minimal stimulation on Treg cells during phase I human clinical trial on advanced or metastatic solid malignancies (71). To enhance anticancer effects, NKTR-214 was synergized with checkpoint inhibitors such as anti-programmed death protein-1 (PD-1) and anti-cytotoxic T-lymphocyte associated protein 4 (CTLA-4), resulting in a drastic reduction of intratumorally Treg cells and an increase in CD8^+^ T cell response without affecting Treg cells in peripheral blood (72). However, the synergic effects of NKTR-2214 with CAR-NK cell therapy still require further investigation.

Apart from using IL-2 to boost NK cells, IL15 is another cytokine that can improve NK cell activation and persistence. Christodoulou et al. demonstrated that anti-CD123-2B4-CD3 CAR-NK was able to increase NK cell persistence by revealing high proliferative and activated transcriptomic NK cell signatures and enhance anti-tumor ability against acute myeloid leukaemia (AML) *in vitro* and *in vivo* when co-expressed with secretory IL-15 (sIL-15) (73). However, this CAR-NK/sIL-15 should be continuously monitored as it possesses cytokine-related systemic toxicity (73). Moreover, NK-92 cell lines transduced with anti-CD19-IL15/IL-15Rα showed robust cytotoxicity against B cell malignancies in both *vitro* and *vivo* and continuously expanded without IL-2 for 21 days (74). Currently, only five clinical trials regarding CAR-NK immunotherapy with IL-15 are listed. However, three clinical trials focusing on cord blood NK cells (NCT06066424, NCT05922930, NCT05703854) and one clinical trial aiming to produce CAR-NK-T cell (NCT05487651) are still in recruiting phase. Nevertheless, one among five clinical trials has been withdrawn due to unknown reasons (NCT03579927).

In addition, IL-21 also shows promising NK cell activation, proliferation, and expansion. Initially, the K562-mIL21 feeder layer is implemented to culture NK cells. However, recent research shows other feeder layers can be used as they promote better expansion for NK cells. Preclinical data from Ojo et al. proved that NK cells cocultured with overexpressing membrane-bound IL-21 feeder layer established from OCI-AML3 cell lines were able to proliferate and expand with robust cytotoxicity against sarcoma and leukaemia *in vitro* and *in vivo* (75). IL-21 activates the STAT3/c-myc pathway in NK cells, thus increasing NK cell proliferation and metabolism (75). An alternative approach such as using an irradiated 221-mIL-21 feeder layer also showed superior advantages over to K562-mIL21 feeder layer owing to their ability to expand memory-like NK cells with less differentiated phenotypes (76). Moreover, human peripheral NK cells can be transduced with CAR-NK-IL21 to promote higher NK cell expansion. He et al. demonstrated that CAR-NK cells co-expressing IL-21 possessed higher expansion fold and produced greater cytotoxic IFN-γ and TNF-α release compared to CAR-NK cells with IL-15 co-expression against CD-19 lymphoma (77). Furthermore, the effort to improve the K562 feeder layer also provides great insight into enhancing NK cell expansion. Zhang et al. found that membrane-bound IL-21 transgene expression followed by co-expressing membrane-bound IL15/IL-15Rα on K562 feeder cells would enhance NK cell yield and anticancer properties (78). At present, there are no clinical trials on employing IL-21 in CAR-NK cell therapy.

Adopting IL-18 to stimulate NK cells is another strategy to increase NK cell persistence. By supplying IL-18 to NK cells, NK cells increased their proliferation and altered into antigen-presenting phenotype, thus mediating T cell responses to target lung cancer cells as shown by Senju et al. (79). Moreover, Gang et al. recently stimulated NK cells with IL-12, IL-15, and IL-18 to induce memory-like NK cells before transducing them into CAR-NK cells (80). Their work showed enhanced persistence and anticancer effect against AML which resists NK cell-based therapy (80). Similar results were also obtained when NK cells treated with the combination of IL-12, IL-15, and IL-18 were then used for manufacturing CAR-NK cells against CD19 of B cell cancers (81). As a result, cancer progression was suppressed along with prolonged survival of CD19 positive tumor-bearing mouse model (81). Nevertheless, none of the clinical trials show combinational therapy of various cytokines to enhance NK cell persistence for now.

### 5.2 Targeting NK cell receptors to improve CAR-NK cell therapy

Activating and inhibitory receptors of NK cells play an important role in augmenting the role of NK cells in cancer elimination. Li et al. showed that incorporation of activating receptors such as natural killer group 2 member D (NKG2D) in the transmembrane region of CAR structure and 2B4 as a co-stimulating domain was able to enhance the cytotoxicity of CAR-NK cells, thus demonstrating the highest efficacy to eradicate cancer cells compared to other CAR structures which did not use NKG2D (82). However, 2B4 cannot be used alone and should bind with CD3 for full CAR-NK cell activation (29). Parihar et al. also demonstrated that CAR-NK cells which incorporated NKG2D in their CD3 able to target NKG2D ligands on MDSCs without interfering NKG2D ligands on normal cells (83). Similarly, enhanced cytotoxicity and reduced cancer cell numbers were attained by fusing the activating receptor, NKG2D with DAP12 in CAR structure (84).

Furthermore, the CD16 activating receptor is another choice of interest to boost CAR-NK cell efficacy. Modification on activating receptors such as CD16 is needed to avoid CD16 from being cleaved by metalloproteinase, ADAM17 which is released by cancer cells (29). By implementing CRISPR technology, the ADAM17 gene was knocked out from CAR-NK cells and the results showed enhanced cytotoxicity *in vitro* and *in vivo* cancer models (85). Similarly, non-cleavable CD16a-hiPSC-NK cells created by Zhu et al. via mutation demonstrated improved survival for mouse-bearing lymphoma (86).

Reducing inhibitory signals on NK cells is another approach to increase the efficiency of CAR-NK cell therapy. A human immunoglobulin (Ig) G4 monoclonal antibody known as IPH2101 was implemented clinically to inhibit inhibitory signals on NK cells by targeting killer cell immunoglobulin-like receptors (KIRs) such as KIR2DL-1, KIR2DL-2, and KIR2DL-3 on NK cells in multiple myeloma patients (87). Conversely, IPH2101 shows unresponsive to NK cell activation due to the loss of KIR2D on NK cells from the trogocytosis event, thus leading to the termination of phase II clinical trial (88). Owing to this problem, a phase I clinical trial using another anti-KIR antibody, so-called IPH2102 (lirilumab) which is derived from IPH2101 was implemented to unlock NK cell inhibition without having dose-limiting toxicity (DLT) (89, 90). In addition, combinational therapy of IPH2102 with other therapeutic agents has been performed recently. For instance, IPH2102 was used to inhibit inhibitory signaling of NK cells by preventing inhibitory KIRs from binding to HLA-C of cancer cells, thus activating ADCC mechanisms to kill the cancer cells that had been marked with cetuximab during head and neck immunotherapy (91). IPH2102 was also coupled with other immune checkpoint inhibitors such as nivolumab and ipilimumab to synergize stronger anticancer effect towards advanced solid malignancies in a clinical trial (NCT01714739). Therefore, more studies regarding anti-KIR antibodies coupled with other therapies such as CAR-NK cells should be performed.

In addition, CD94 or NK group 2 member A receptor (NKG2A) is important in inhibiting NK cells by binding to its ligand, HLA-E on cancer cells. Monalizumab is used to target NKG2A, thus activating NK cells to exhibit cancer-killing ability (92). Moreover, monalizumab synergized with cetuximab to target head and neck squamous cell carcinoma, resulting in remarkable antic-cancer effects with a 31% response rate among patients in phase II clinical trials (93). Recently, monalizumab coupled with MEDI5752 which is a bispecific monoclonal antibody targeting checkpoint inhibitors such as PD-1 and CTLA-4 is a promising strategy to target highly immunogenic cancers in MONAMI clinical trial (NCT06152523). Nonetheless, the combination of monalizumab with trastuzumab did not achieve targeted clinical responses although the breast cancer respondents were free from DLT in the MIMOSA trial (NCT04307329), indicating that the low respondent number and scarcity of tumor infiltrating lymphocytes may inhibit anti-NKG2A therapy (94). Therefore, CAR-NK cell therapy coupled with anti-NKG2A antibodies is an urgent topic that requires further investigation to improve the overall survival of cancer patients.

NK cell engagers are another promising strategy to improve the efficacy of CAR-NK cell therapy. There are two types of NK cell engagers: BiKE (bispecific killer cell engager) and TriKE (trispecific killer cell engager) which bridge NK cells with cancer cells more effectively, thus enhancing the efficacy of NK cells to eliminate cancer cells. Various activating and inhibitory receptors on NK cells have been targeted by NK cell engagers to improve NK cells in cancer eradication, including CD16a, CD160, CD96, NKG2A, NKG2D, NKG2C, Nkp30, Nkp46, Nkp80, KIR2DS/KIR3DS, KIR2DL/KIR2DS, DNAM-1, 2B4, PD-1, IL-2/IL-15, TIGIT, and TIM-3 (95). Nonetheless, each receptor possesses pros and cons when targeted with NK cell engagers. Nikkhoi et al. showed BiKE targeting CD16 of NK cells and HER2 antigen on cancer cells was able to generate a high level of ADCC compared to conventional antibody, trastuzumab (96). Remarkable results of using BiKE are also shown in hematological malignancies (97). Moreover, TriKE armoring anti-CD16, anti-CD19, and IL-15 enhanced cancer cell lysis and cytotoxicity against CD19-positive tumors (98). In addition, anti-B7H3 CAR T cells synergized anticancer effects with BiKE which target CD16 of NK cells, thus providing attractive insight into the use of NK cell engagers with CAR-NK cell therapy (99). However, there is still lacking preclinical and clinical data on using NK cell engagers with CAR-NK cells. The idea of using NK cell engagers to bridge CAR-NK cells with other therapeutic agents such as antibodies may provide better cytotoxicity than BiKE or TriKE monotherapy, but this concept yet to be investigated.

### 5.3 Overcoming immunosuppressive tumor microenvironment

Cancer, especially solid tumors, consists of heterogenous cell populations including immune cells which are suppressed and anergic in complex tumor microenvironments. This immunosuppressive tumor microenvironment is not only hypoxic, but it also possesses altered metabolism which supports the rapid proliferation of cancer cells.

To revitalize NK cells from the suppressive tumor microenvironment, SX-682 which is an inhibitor targeting chemokine receptor 1/2 (CXCR1/2) showed promising inhibition towards suppressive myeloid-derived suppressor cells (MDSCs), thus preventing them from infiltrating into tumor microenvironment of head and neck cancer and deactivating NK cells (100). However, the synergic effects of SX-682 with CAR-NK cell therapy should be further investigated as none of this study has been reported so far. Furthermore, cancer cells accumulate high levels of adenosine triphosphate (ATP) which in turn dephosphorylated into adenosine by CD73 of MDSCs can eventually inhibit NK cells (101). Chambers et al. transduced NK cells with anti-CD73 single chain fragment variable (scFv) CAR and successfully eliminated overexpressed CD73 lung cancer cells under hypoxic condition (101). Moreover, a drug such as nintedanib reduced IL-6 produced by cancer-associated fibroblasts (CAFs), thus enhancing the cytotoxicity of CAR-NK cells against mesothelin-positive cancer by increasing NK cell activating receptors (102).

In addition, inhibition of monocarboxylate transporter 4 (MCT4) which regulates lactate secretion and exchange led to the activation of cytotoxic NK cells to kill immunosuppressive breast cancer cells (103). In addition, high proliferative cancer cells use up the nutrients in the tumor microenvironment, leaving a nutrient-deficient hostile environment for immune cells, thus suppressing immune reactions. Therefore, Nachef et al. suggested engineering CAR-NK cells with amino acid transporters such as SLC1A5, SLC3A2, and SLC7A5 to improve amino acid uptake for CAR-NK cells and prevent them from being suppressed by nutrient-deficient tumor microenvironment (104). Nonetheless, there are no studies focusing on using CAR-NK cells directly to target MCT4 of cancer cells or enhancing CAR-NK cells with amino acids transporters currently. Furthermore, suppressive immune cells such as Tregs, tumor-associated macrophages (TAMs) and MDSCs secrete TGF-β to impede NK cell function. To tackle immunosuppressive TGF-β in the cancer microenvironment, Chaudhry et al. demonstrated that CAR-NK targeting B7H3 of glioblastoma enhanced tumor lysis even with the presence of immunosuppressive TGF-β when co-expressing this CAR-NK with TGF-β dominant negative receptor (DNR) (105). However, the *in vivo* model was not investigated during this study, thus more preclinical data are required to support the efficacy of CAR-NK/TGF-β DNR in targeting immunosuppressive cancers.

In addition, cancers which overexpress immune checkpoints such as PD-L1 possess poor prognosis and suppressive behavior towards NK cells in the tumor microenvironment. This could be targeted by incorporating anti-PD-L1 scFv directly into NK cells. Liu et al. proved that tumor regression in the humanized nasopharyngeal cancer (NPC) mouse model was achieved by designing CAR NK cells harboring anti-PD-L1 properties and synergized anticancer effects with a checkpoint inhibitor, nivolumab (106). Anti-PD-L1 CAR-NK was combined with anti-PD-1 and IL-15 superagonist, N-803 to boost NK cell ability in killing carcinoma *in vivo* while reducing T cell exhaustion and mediating T cell responses via IFN-γ release which is induced by IL-15 (107). Moreover, the combinatorial therapy of implementing anti-PD-L1 CAR-NK with anti-PD-1, pembrolizumab and N-803 is currently recruiting in the clinical trial (NCT04847466) to target refractory gastric and head and neck malignancies. From another perspectives, Lu et al. designed a chimeric costimulatory converting receptor (CCCR) in the structure of CAR to alter negative signals of PD-1 to activating signals, thus modulating, and hindering immunosuppressive tumor microenvironment via this novel CAR-NK cells (108). Furthermore, another immune checkpoint, B7H3 is a promising candidate to be targeted. Yang et al. demonstrated that anti-B7H3 CAR-NK cells increased granzyme and perforin secretion to lyse non-small lung cancer cells (109). In addition, immune checkpoint, HLA-G of cancer cells can be targeted by CAR-NK to inhibit immunosuppression. Jan et al. proved that CAR-NK cells targeting HLA-G were able to induce cytotoxicity against solid malignancies after pre-treating with chemotherapy (110). Cancer cells sensitized by chemotherapy highly expressed their HLA-G, thus promoting better activation and cancer antigen recognition for CAR-NK cells (110).

### 5.4 Improving CAR-NK cell trafficking and infiltration

Proinflammatory cytokine such as IL-8 is produced by cancer cells during tumor development. Therefore, IL-8 becomes an attractive candidate to mediate the infiltration of CAR-NK cells into complex and immunosuppressive tumor microenvironment. By designing CXC motif chemokine receptor (CXCR) 1 with CAR construct in NK cells, these CXCR1/CAR-NK cells promoted effective trafficking and peritoneal infiltration into the ovarian cancer microenvironment (111). In hematological malignance, mRNA of CXCR4 and anti-B cell maturation antigen (BCMA) CAR were electroporated in NK cells to increase homing ability to bone marrow of multiple myeloma xenograft, thus enhancing cytotoxicity of CAR-NK cells and remarkably reducing tumor burden (112).

Chemokine receptor (CCR) is significant recruiting NK cells into the tumor microenvironment. Schomer et al. proved that CCR7-modified anti-CD19 CAR-NK cells effectively controlled the growth of CD19-positive lymphoma in mouse models by enhancing the infiltration of CAR-NK cells towards chemokine ligands, CCL9 which produced by lymphoma cells (113). Furthermore, chemokines such as CXCR2 also improved the trafficking and infiltration of NK cells into the tumor microenvironment of renal cancer, thus providing great insight into genetically engineering CXCR2 on CAR-NK cells (114). Nonetheless, more studies should be conducted to ensure the induced chemokine will not cause negative effects on cancer patients or promote tumorigenesis.

### 5.5 Overcoming immune escape

Cancer cells which shed their MHC class I chain-related protein A (MICA) and protein B (MICB) can exhibit immune escape properties and ultimately promote rapid cancer progression. Therefore, an antibody targeting MICA α3 was synthesized to prevent the proteolysis of MICA and MICB from cancer cells, thus allowing the binding of NK cell activating receptor, NKG2D with MICA/B to activate cytotoxic NK cells (115). There are also various approaches to prevent immune escape by improving antigen recognition by CAR-NK cells. As mentioned, iDuo NK cells targeting anti-CD19 and CD20 improved NK cell recognition, thus preventing immune escape (19). Similarly, dual CAR-NK cells targeting BCMA and GPRC5D also prevented immune escape for multiple myeloma cells which downregulates BCMA antigen (21).

**Figure 2:**
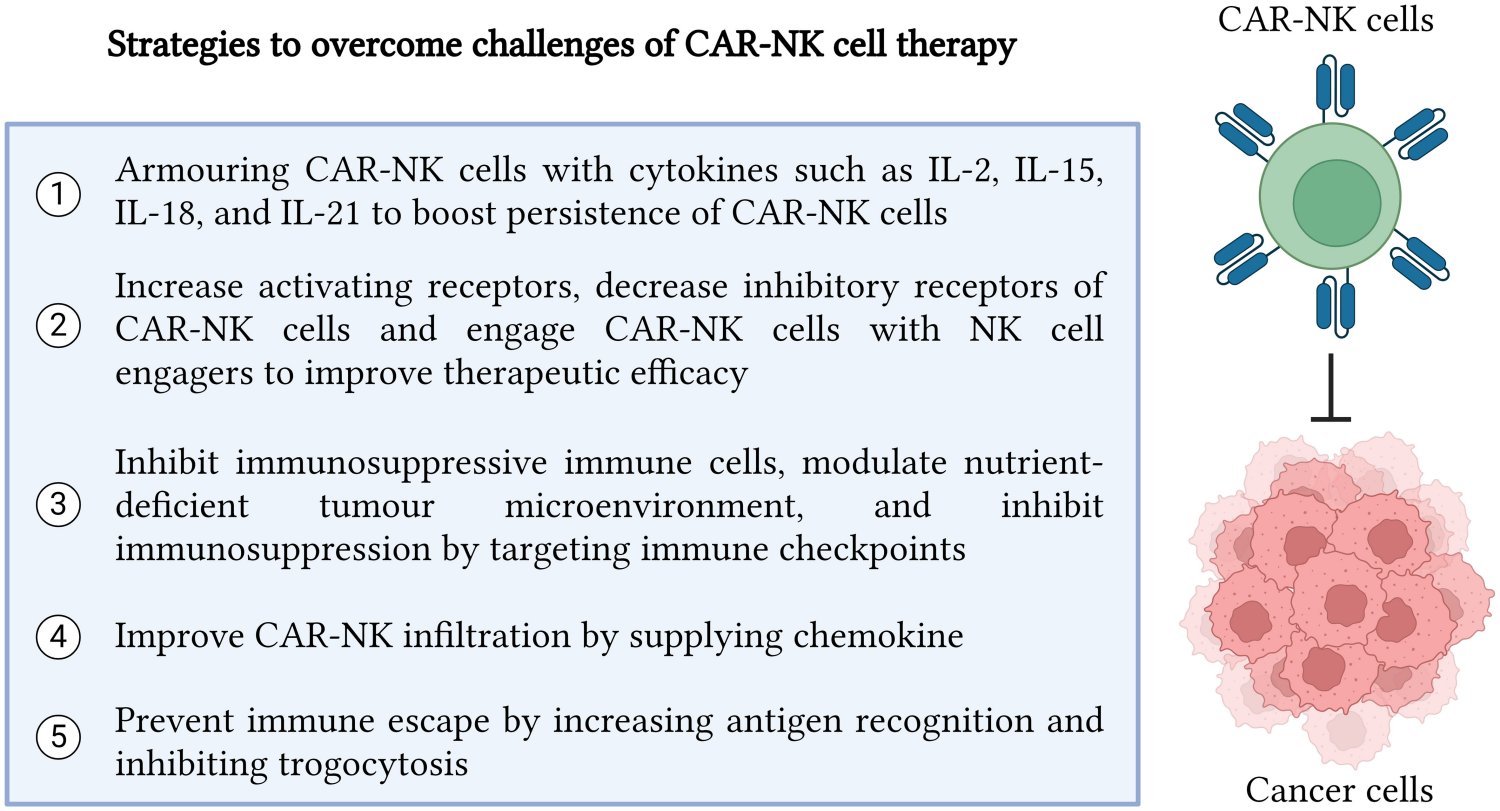
Strategies to overcome challenges in CAR-NK cell therapy. In summary, there are five major strategies which can be implemented to enhance CAR-NK cell efficacy in recognizing and killing cancer cells precisely. The five strategies include cytokine armoring, NK cell receptor modification, inhibition of immunosuppressive tumor microenvironment, improvement in CAR NK infiltration and trafficking, and immune escape prevention. Created with BioRender.com.

Moreover, fratricide of NK cells which is induced by trogocytosis event becomes a major challenge for CAR-NK cell therapy as it will promote immune escape and eventually lead to cancer relapse. To resolve this issue, Li et al. developed a dual CARs system which comprised of a CAR to target cancer antigen and an inhibitory CAR to send inhibitory signals to inhibit CAR-NK cells from targeting NK cells which present trogocytic antigen (TROG) due to trogocytosis event (25). Therefore, fratricide of NK cells can be prevented by using dual CAR-NK cells and eventually prevent cancer cells from immune escape. Apart from developing dual CAR-NK cells, the use of latrunculin A (an actin inhibitor) can prevent trogocytosis since actin is important for the exchange of cancer antigens from the cancer cell surface onto NK cells (116). Moreover, the upregulation of cholesterol 25-hydroxylase (CH25H) inhibits lipid membranes from fusing. Therefore, it can be targeted by SUMOylation inhibitors such as TAK981 to prevent trogocytosis (116). Furthermore, Lu et al. proved that co-expressing CH25H in CAR structure successfully inhibited trogocytosis and improved the tumoricidal ability of CAR-T cells (117). Therefore, it will be very attracting to leverage the combinational effect of armoring CH25H in CAR-NK cells to prevent trogocytosis while exploring their synergic role in cancer elimination. In addition, immune escape could be reduced when the CD38 gene from NK cells was knocked out to reduce fratricide before transducing the CD38 knockout NK cells with anti-CD38 CAR to target CD38 of AML (118). Similarly, CD38 knockout dual CAR-NK cells targeting BCMA antigen were able to prevent trogocytosis and maintained high cytotoxicity against multiple myeloma cells (20). The strategies to overcome challenges in CAR-NK cell therapy are illustrated in **Figure 2**.

### 5.6 Reverting NK Cell Exhaustion

NK cell exhaustion can be reduced by targeting inhibitory signals induced by KIRs, PD-1, TIGIT, NKG2A, and TIM-3 of NK cells (62, 119). Moreover, various alkylating agents, proteasome inhibitors, histone deacetylation inhibitors, hyperploidy-inducing agents, and glycogen synthetase kinase-3 can be implemented to upregulate the activating receptor, NKG2D on NK cells (120). Furthermore, the activation, proliferation, and persistence of NK cells or CAR-NK cells can be boosted by supplementing various cytokines such as IL-2, IL-12 and IL-18 as mentioned previously in the section 5.1 to decrease NK cell exhaustion. However, there are still lack of studies on NK cell exhaustion on CAR-NK cell therapy. This may be due to CAR-NK cell therapy is still considered as a new promising therapy which requires more on-going preclinical and clinical data to evaluate the effectiveness and persistence of CAR-NK cells in long term studies.

To date, there are studies on the potential causes and strategies to overcome CAR-T exhaustion which can serve as an insight for CAR-NK cell therapy. CAR-T cells experience exhaustion probably due to the induction tonic signaling which continues stimulating T cells to respond and activate even without any antigen stimulation (121). Moreover, the structure of CAR-T influences T cell exhaustion. As shown by Saren et al, the different amino acid sequences in complementary determining region (CDR) could result the clustering of CAR structure, thus leading to antigen-independent tonic signaling in CAR-T cells (122). Conversely, Long et al. proved that the cause of T cell exhaustion in self-activating GD2.28z CAR-T cells was due to the clustering of framework region (FR) instead of CDR regions (123). Therefore, it is still unclear to conclude the main reason of CAR-T cell exhaustion from its structure.

CAR-T cell exhaustion is associated with upregulation of inhibitory signals. Therefore, various strategies targeting immune checkpoints or inhibitory receptors such as PD-1, CTLA-4, TIM-3, TIGIT, and LAG-3 of T cells have been extensively studied to remove T cell exhaustion (124, 125). Moreover, epigenetic activation of c-Jun axis, knockdown of transcription factors such as T-bet and Eomes, and downregulation of TOX/NR4A expression can be used to reduce T cell exhaustion (124). Similarly, cytokines can be supplied to activate and maintain T cell persistence (121). Nevertheless, there are less studies on the phenotypes and exhaustion gene profiles of CAR-NK cells, thus this part remains unknown and yet to discover.

## 6 Current status of CAR-NK cell therapy

To date, there are 49 clinical trials on CAR-NK cell therapy listed in ClinicalTrials.gov. The details for each clinical trial are tabulated in **Supplementary Table 1**. Among the clinical trials listed, 36 clinical trials focus on CAR-NK cells in treating hematological malignancies while only 13 CAR-NK clinical trials target solid malignancies. Therefore, more clinical studies should be conducted on solid tumors to evaluate the efficacy of CA-NK cells on different solid malignancies. Moreover, the safety of cancer patients after treating with CAR-NK cell therapy is the utmost important clinical assessment which requires prolonged monitoring.

Moreover, most NK cell sources are from allogenic NK cells although not mentioned in clinical trials. There are other sources of NK cells used such as NK-92 cell line and its derivatives, umbilical cord blood, patient peripheral blood, and induced pluripotent stem cells (iPSCs). Currently, most of the CAR-NK clinical trials are still recruiting, thus highlighting this CAR-NK cell therapy is a new promising immunotherapy to target various cancers.

## 7 Future recommendations on CAR-NK cell therapy

In future, radiotherapy can combine with CAR-NK cell therapy to provide effective treatment for cancer patients. He et al. proposed that using radiotherapy before CAR-NK cell therapy could be a promising strategy to increase the trafficking, infiltration, and recognition of NK cells in tumors (126). However, more studies are required to seek out the optimal dose, duration, and sequence of combinational therapy, as well as to overcome immunosuppression mediated by radiotherapy (126). Moreover, chemotherapy can be used prior to CAR-NK cell therapy to increase cancer antigens released from tumor lysis, thus improving antigen recognition and activation of CAR-NK cells in tumor (127). Chemotherapy can also enhance CAR-NK cell therapy by killing suppressive immune cells such as MDSCs and Tregs in the tumor microenvironment (127). Furthermore, the combination of CAR-NK cell therapy with oncolytic virus also offers better anticancer results. For instance, anti-EFGR CAR-NK cells synergized with oncolytic virus expressing IL-15/IL15Rα sushi domain fusion protein enhanced the persistence and infiltration of CAR-NK cells and suppressed the growth of glioblastoma *in vitro* and *in vivo* (128).

In another perspective, drug-conjugated nanoparticle shows remarkable tumoricidal effects towards cancer cells. To explore the possible anticancer effects of combining drug-conjugated nanoparticles with cell-based therapy, CAR-NK cells were conjugated with cross-linked multilamellar liposomal vesicles (cMLVs) carrying chemotherapy agent, paclitaxel (PTX) to target cancer cells (129). This combinational therapy showed enhanced PTX drug delivery to cancer cells via CAR-NK cells as drug carriers and successfully eradicated cancer cells, thus highlighting the synergistic effects of nanoparticles with CAR-NK cell therapy (129).

There are also more efforts combine CAR-NK cells with monoclonal antibodies, especially immune checkpoint inhibitors. One of the examples is using PD-L1 monoclonal antibody to treat prostate cancer together with CAR-NK cells targeting prostate-specific membrane antigen (PSMA) (130). Searching for other possible combinational therapies is important to strengthen the efficacy of CAR-NK therapy against cancer cells. An approach of combining CAR-NK cells targeting epithelial cell adhesion molecule (EpCAM) with tyrosine inhibitor, regorafenib was able to boost the efficacy of CAR-NK cells against colorectal cancer (131). Moreover, romidepsin which inhibits histone deacetylase was synergized with anti-CD20 CAR-NK cells to inhibit rituximab-resistant Burkitt lymphoma with enhanced cytotoxicity induced (132). Interestingly, romidepsin increased the expression of MICA/B on lymphoma cells to activate CAR-NK cells via NKG2D activating receptor (132).

To improve the efficacy of CAR-NK cell therapy, the idea of combining CAR-NK cells with CAR-T cells has been proposed. This new concept was demonstrated by Li et al. in which CAR-T cells improved the proliferation of CAR-NK cells while CAR-NK cells reduced the risk of CRS (133). In combination, both CAR-T and CAR-NK cells showed inhibition towards cancer progression and relapse (133). Nonetheless, the side effects of implementing both CAR-T and CAR-NK therapy are still unknown for clinical study, thus this combinational therapy is still yet to be explored more.

CAR-NK cell therapy is considered a new paradigm shift for cancer immunotherapy. Future efforts on optimizing robust and tumor-specific CAR structure should be conducted actively to overcome the challenges of CAR-NK cell therapy. Moreover, combining CAR-NK cells with other therapeutic agents should be continuously explored. Currently, studies on vaccines with CAR-T cells have boosted the efficacy of CAR-T cells in eradicating cancer cells (134). Therefore, this combination provides a promising insight to implement this strategy on CAR-NK cells. Moreover, more clinical data are required to ensure the safety profile of CAR-NK cells in cancer patients.

## 8 Food and drug administration guidelines on CAR-NK cell products

The safety and quality of CAR-NK cell products require further monitoring and assessments. Therefore, the FDA has released guidelines to facilitate the production of CAR products so that the products will adhere to Good Manufacturing Practices (GMP). To summarize, the FDA has recommended several guidelines regarding general CAR design and development, chemistry, manufacturing, and control (CMC) of CAR products, as well as clinical and non-clinical recommendations. All the guidelines are tabulated in **Table 2**.

**Table 2:** FDA Guidelines on CAR Products.

Aspects	Category	Recommendations	Reference
General CAR design and development	CAR construct	Design highly specific and safer CAR construct with minimal risk of on target off tumour toxicity	(135)
	Vector used	Use well characterized and safer vector to avoid insertional mutagenesis	
	Cellular starting material	Use healthy T or NK cells and perform leukapheresis effectively	
	Fresh or cryopreserved CAR products	Check the shelf life of fresh CAR-T or CAR-NK cells before infusing Check the stability, viability, and quality of cryopreserved CAR-T or CAR-NK cells during shipping, receipt, storage, and preparation for infusion	
Chemistry, manufacturing, and control (CMC) of CAR products	Vector manufacturing and testing	Conduct robust manufacturing and testing procedures Maintain vector quality and sterility	
	Cellular starting material	Standardize the collection, handling, and testing protocols for cellular starting material to minimize contamination	
	CAR-T/NK cell manufacturing and testing	Validate manufacturing process to ensure consistency and reproducibility Perform comprehensive analytical testing for final CAR product	
	Dealing changes and comparability	Develop procedures for managing changes in manufacturing and testing Perform comparability studies for major alterations in manufacturing	
	Single site or Multisite	Perform routine quality control and consistency checking	
Nonclinical Recommendations	Cellular Component	Characterize T cell or NK cell phenotype and functionality after CAR modification.	
	*In vivo* testing	Conduct *in vivo* studies to assess efficacy and safety of CAR products	
	CAR modifications	Evaluate the safety and efficacy of CAR modifications	
Clinical Recommendations	Study population	Study tissue-agnostic approaches for broadly applicable CAR products. Select target population carefully especially vulnerable folks and pediatrics	
	Treatment plan	Optimize dose for starting, escalation, repeating, staggering, and bridging therapy as well as developing contingency plan for treatment failure	
	Pharmacological considerations	Assess pharmacokinetics, pharmacodynamics, immunogenicity, and immune response of CAR products.	
	Safety evaluation and monitoring	Evaluate toxicity of CAR products and implement monitoring strategies for adverse events	
	Persistence of CAR product and long term follow up	Monitor CAR-T/NK cell persistence and efficacy with long term follow up	

## 9 Conclusion

In conclusion, CAR-NK cell therapy is a new promising immunotherapy to target cancer cells due to it possesses robust cytotoxicity against cancer cells without eliciting side effects such as GvHD, CRS and neurotoxicity which are present in CAR-T cell therapy. Advancements have been made to create better CAR-NK therapy by either enhancing CAR structure or improving CAR-NK production. Enhancing CAR structure can be performed by revolutionizing the generation of CAR and increasing antigen recognition of CAR structure. While improvements on CAR-NK production can be done by exploring various NK cell sources, improving CAR gene delivery system, and enhancing CAR transduction or transfection into NK cells.

However, CAR-NK cells therapy still possesses some obstacles such as lack of persistence in NK cells, ineffective CAR-NK cell therapy, immunosuppressive tumor microenvironment, ineffective CAR-NK cell trafficking and infiltration, and immune escape. To resolve these limitations, various strategies are implemented to target the challenges respectively. Armoring cytokines to boost NK cell persistence, targeting NK cell receptors to improve efficacy of CAR-NK cells, engaging CAR-NK cell with NK cell engagers, targeting immunosuppressive tumor microenvironment to revitalize CAR-NK cells, supplying chemokine to improve CAR-NK cell infiltration, and reducing trogocytosis to prevent immune escape are the strategies used to overcome challenges of CAR-NK cell therapy. Furthermore, combinational therapy for CAR-NK cells with other therapeutic agents is the future direction of adoptive cell-based immunotherapy. Overall, clinical trials of CAR-NK therapy are moving towards solid tumor targeting. Thus, more studies should be performed to generate better and safer CAR-NK cell therapy. CAR-NK cell therapy can be tailored to become “off-the-shelf” personalized medicine which can accommodate to treat different cancers in future. In addition, FDA guidelines on CAR-NK cells should be implemented to ensure the safety and quality of CAR-based therapy.

## Author contributions

JK: Conceptualization, Writing – original draft, Writing – review & editing. MS: Writing – review & editing. HV: Writing – review & editing. MR: Writing – review & editing. VB: Writing – review & editing. ST: Funding acquisition, Supervision, Writing – review & editing. GT: Conceptualization, Funding acquisition, Supervision, Writing – review & editing.
